# Plant growth promotion mechanisms of bacteria isolated from a long-term reclaimed smelter waste deposit

**DOI:** 10.1038/s41598-025-21980-w

**Published:** 2025-10-30

**Authors:** Sylwia Siebielec, Małgorzata Martyna Woźniak, Artur Nowak, Grzegorz Siebielec, Monika Kozieł, Piotr Sugier, Jolanta Jaroszuk-Ściseł

**Affiliations:** 1 Department of Microbiology, Institute of Soil Science and Plant Cultivation—State Research Institute, Czartoryskich 8, 24-100 Pulawy, Poland; 2 Department of Industrial and Environmental Microbiology, Faculty of Biology and Biotechnology, Maria Curie-Skłodowska University, Akademicka 19, 20-033 Lublin, Poland; 3 Department of Soil Science and Environmental Analyses, Institute of Soil Science and Plant Cultivation—State Research Institute, Czartoryskich 8, 24-100 Pulawy, Poland; 4 Department of Botany, Mycology and Ecology, Institute of Biological Sciences, Maria Curie-Skłodowska University, Akademicka 19, 20-033 Lublin, Poland

**Keywords:** Bacteria strains, Phosphorus solubilizing bacteria, Plant growth promotion, PGPR, Plant stress tolerance, Smelter wasteland, Microbiology, Ecology, Environmental sciences

## Abstract

The use of beneficial bacterial strains is proposed as a nature based practice to support sustainable crop production. Strains exposed to extreme environmental stress may have developed robust stress resistance and the capacity to enhance plant growth under unfavorable conditions. Our study provides the new aspect in characterising bacteria from polluted soil. The novelty of our study was isolation of bacteria from a long-term contaminated site and their testing for plant growth promoting mechanisms. The aim of this research was to characterize bacterial strains, collected from the root zone of grasses growing in a heavily polluted smelter wasteland reclaimed 25 years ago using sewage sludge and by-product lime. Their capability to enhance plant resistance to stresses has not been widely assessed. The activity of the strains was assessed based on mechanisms associated with nutrient uptake: phosphate solubilization, ability to fix atmospheric nitrogen (N), ability to synthesize indole-3-acetic acid (IAA)-like compounds, and mechanisms linked to plant stress tolerance: ACC deaminase production, polysaccharides and biofilm development. Metabolic profiling of the strains was performed. Most strains tested in this study exhibited a range of plant growth promotion mechanisms. All strains solubilized phosphates with medium to high intensity, 14 of 15 isolates produced IAA up to 60 µg/mL, all fixed N from 15.85 to 50.00 mg/ml after 72 h. Thirteen strains survived freeze-drying. Our study enabled clustering bacterial strains with capability to perform certain groups of processes. Strains intensively fixing N in general were also able to intensively produce IAA but rather were not efficient producers of extracellular polymeric substances (EPS). IAA production was negatively correlated with 1-aminocyclopropane-1-carboxylic acid deaminase (ACC) deaminase activity and average carbon utilization intensity. All three strains selected for the pot study (*Burkholderia* sp., *Pseudomonas caspiana, and Phyllobacterium* sp.) confirmed the effectiveness in promoting wheat growth both at optimal and low soil moisture. The study shows that 25-years reclaimed smelter wastelands are reservoirs of PGPR strains potentially useful for developing biofertilizers enhancing plant growth and resistance to environmental or climatic stresses in agriculture.

**Supplementary Information:**

The online version contains supplementary material available at 10.1038/s41598-025-21980-w.

## Introduction

Plant Growth Promoting Rhizobacteria (PGPR) are beneficial microorganisms that colonize the rhizosphere and directly or indirectly enhance plant growth and development, and crop yields^1–8^. The effects of PGPR on plant growth has been documented for wide representation of soil conditions^9–12^. The bacteria-based bioinoculants not only affect the cycling of nutrients, but also protect the plant against pests and diseases, promote the root system and decompose harmful chemicals^3,13^. Other beneficial mechanisms of PGPR include: decomposition of organic matter, formation of biofilm, synthesis of plant hormones such as indole-3-acetic acid (IAA), cytokinin, and gibberellin, solubilization of micronutrients, that might contribute to the alleviation of abiotic and biotic stresses^14–16^. Importantly, the same microorganism can perform several of the above-mentioned functions. Such synergistic effects accelerate its beneficial effect on plants^17–19^.

Role of PGPR and mechanisms involved in alleviating plant abiotic stresses have been studied and discussed in literature^20^. Navarro-Torre et al.^21^ provided extensive overview of microbial mechanisms supporting legume growth under environmental stressors, such as high temperatures, drought, salinity, and soil pollution. The mechanisms listed include phosphate solubilisation, N fixation, phytohormone production, siderophore production, ACC deaminase activity and some indirect mechanisms like production of antibiotics. Orozco-Mosqueda at el.^22^ paid attention to techniques leading to develop PGPR bioformulations, their forms (power, liquid) and importance of bacteria selection and application way. Early stage application methods are considered as effective (on seeds or seedlings). It is also important that some bioinoculants can have dual action, for example direct stimulation of plant growth and at the same time, antagonizing pathogens and stimulating plant defense.

There are studies available indicating that for certain purposes (application to promote plant growth in contaminated soils) there is a need to screen for bacteria resistant to environmental stresses (for example arsenic content, high salinity) to increase the likelihood of their effectiveness under chemical stress conditions^23,24^.

From the review of available literature it is clear that PGPR can promote plant growth under stress conditions. Therefore, it is very important to screen for most efective bacteria that would be also well adapted to the stress conditions. We can assume that type of soil environment from which the bacterial strains are obtained strongly affects the strain effectiveness. Smelter wastelands represent specific conditions for microbial growth, namely extreme contamination with heavy metals, drought, deficiency of nutrients, salinity. Therefore, we assumed that soils developed from former smelter wastes might provide bacterial strains that are adapted and active under limited soil moisture, low availability of nutrients, salinity, chemical stress related to contamination. Previous studies have shown non-typical structure of the microbiome and its significant role in the colonization of waste piles by plants and their adaptation to the harsh conditions occurring there^25,26^.

Potential of microorganisms isolated from contaminated sites to exhibit plant growth promoting mechanisms is an extremely interesting and innovative issue. The bacteria were isolated from the extreme niche, such as long-term reclaimed smelter waste deposit. Although fungi are equally important as a source of microorganisms for developing biofertilizer formulas, we focused our study on bacterial strains since the alkaline conditions of the smelter waste favor development and diversity of bacterial communities.

This research might constitute an initial step in developing stress-resistant bacterial components of biofertilizers dedicated to promoting growth of agricultural crops and remediation plants.

The bacteria were isolated from a smelter wasteland located in Upper Silesia in Poland, on the premises of a closed zinc and lead smelter in Piekary Śląskie (geographic coordinates of the center of the site: 50.364505 N; 18.968960 E) (Figure S1). This area was reclaimed in 1996. The waste deposit consisted mainly of slags from the “Waeltz” metallurgical process, which takes place in furnaces used to enrich ores with low Zn and Pb content. The slags were extremely rich in lead (Pb), zinc (Zn), cadmium (Cd) and arsenic (As)^25,27^. The total contents of Zn, Pb, Cd and As, exceeded in control plots 19,000, 4800, 160 and 1200 mg/kg, respectively^27^. Before remediation, the waste heap was barren, which posed a serious threat to the environment. The remediation protocol involved application of municipal sewage sludge and waste lime as soil amendments, followed by sowing the grass mixture^25,27^. Twenty years after the remediation (year 2016) the following grass species were dominating: *Festuca rubra, Poa pratensis, Festuca arundinacea, Festuca ovina* and *Agrostis capillaris*. The samples wer ecollected from the plots reclaimed with the combination of high rates of sewage sludge and waste lime. The chemical and microbiological characteristics of the smelter wasteland have been previously reported^25^. The topsoil of the site was characterised by alkaline pH (7.6 to 8.3) and still relatively high supply of calcium carbonate. The smeltar waste was also highly saline – the electrical conductivity reached 7.3 dS/m before the site rmediation^27^. The previous studies revealed strongly positive effect of sewage sludge combined with lime on soil biodiversity, abundance of bacteria and biochemical activity of the constructed soil^25^.

The aim of our research was to characterize potential PGPR strains, isolated from the root zone of grass plants growing in an extremely polluted smelter wasteland.

## Materials and methods

### Bacterial sampling, isolation and storage

In this publication, we present results of testing 15 bacterial strains isolated from the reclaimed smelter wasteland. The soil material aimed at isolation of bacteria was collected in 2021 from root zone of grass species constituting relatively compact grass cover: *F. rubra*, *P. pratensis*, *F. arundinacea*, *F. ovina* and *A. capillaris*.

Bacteria were isolated using the soil dilution method on a medium used for phosphate-dissolving microorganisms^28,29^. This medium contains insoluble tri- or bicalcium phosphate as the sole source of P, enabling the detection of phosphate-solubilizing microorganisms (PSM). This technique involves a series of dilutions of the soil suspension onto a sterile Pikovskaya agar medium (PVK). After an appropriate incubation time (7 days) at a temperature of 28 ± 2 °C, the analysis result is read. The formation of a solubilization zone (transparent halo zone) near the growth of microorganisms is considered as a positive result^29,30^. Growing colonies of microorganisms were passed through with reduction culture several times in order to clean them and obtain pure bacterial cultures. Then, clean single colonies were transferred to agar tubes and stored in a laboratory refrigerator at a temperature 4 ± 1 °C at 4 °C on tryptic soy agar (TSA) (Difco Laboratories, Inc., Franklin Lakes, NJ, USA). Bacteria were also lyophilized which helps to maintain their viability during storage. After 48 h incubation at 25 °C, a liquid bacterial culture was centrifuged and then the bacterial sediment was suspended in a sucrose solution. The material was then transferred to lyophilization vials and closed with tight stoppers, and subjected to freeze-drying processes (for 24 h at − 30 degrees, followed by 12 h at -20 degrees, and final 12 h at 20 degrees). Bacterial growth was also confirmed on LB (lysogeny broth) medium (Table S1). A majority of tested isolates showed high or medium growth on all media used. Two isolates did not exhibit growth after freeze-drying. Moreover, these were bacterial strains characterized by lower growth efficiency compared to other strains (Table S1). However, since these strains exhibited substantial activity in further laboratory tests they are presented in the paper. Despite lack of regrowth after freeze-drying they still can be potentially used as biofertilisers since there may be alternative methods to freeze-drying to preserve the viability of these bacterial strains.

### Potential of phosphate solubilization

Phosphate-solubilization was determined qualitatively by plating bacteria on PVK that contains precipitated tricalcium phosphate Ca_3_(PO_4_)_2_. As positive result was considered the formation of a solubilization zone (transparent halo zone) near the growth of microorganisms (Figure S2). The ability of individual microorganisms to solubilize phosphates was ranked on the basis of the solubilization index (SI). The isolates were classified as low (SI < 2), middle (2 < SI ≤ 4), and high solubilizers (SI > 4), following the publication of Berraquero et al^31^.

### Ability to fix atmospheric nitrogen

Content of total N in the examined bacterial cultures after 24, 48 and 72 h of incubation at 28 ºC was analyzed based on the Dumas method^32^. Bacterial isolates were inoculated into 10 ml of N-free liquid medium^33^ in glass bottles and placed in an orbital shaker for 72 h. After each incubation period, 2 ml of bacterial culture was placed in a glass test tube and 2 ml of 1N NaOH was added. The same liquid medium but not inoculated was treated as a control. The test tubes were then placed in a water bath at 80 °C for 1 h. After cooling, the contents of the test tubes were neutralized with 2 ml of 1N HCl. The solutions prepared in this way were poured into glass vials and placed on the Analitik Jena Multi N/C 2100 analyzer in order to determine the total nitrogen content.

### Synthesis of IAA-like compounds

IAA-like compounds production by selected strains with the presence of 0.1% L-Tryptophan (L-Trp), was measured using Salkowski reagent (35% HClO4 50 ml, 0.5 M FeCl3 1 ml)^34,35^. The strains were incubated in triplicate in TSB at 28 °C for 48 h on a rotary shaker. Then, culture supernatants were collected after centrifugation at 10,000 g for 10 min. Non-inoculated control medium was kept for comparison. Two mL of culture supernatant was mixed with 4 mL of Salkowski reagent and allowed to react in darkness at room temperature for 30 min. The development of a pink color indicates the presence of IAA. The measurement was performed in 3 repetitions. The color intensity was quantified colorimetrically at 530 nm. The concentration of IAA produced was measured using a Thermo Fisher Scientific Evolution 60 UV–VIS spectrophotometer.

### ACC deaminase production

ACC deaminase activity was quantified according to the modified Penrose and Glick method, described by Woźniak et al. 2023^36^. This method involves α-ketobutyrate (α-KB) measurement which is produced during ACC hydrolysis performed at 28 °C in DF medium^37^. The medium was supplemented with (NH_4_)_2_SO_4_ (control) or 3 mM ACC (test sample) as the nitrogen source. ACC deaminase activity was normalized per mg protein and therefore expressed as α-KB produced in µmol per mg protein per 1 h. The cultures were incubated for 72 h. Total protein content was estimated using the Bradford method (BioRad, Hercules, CA, USA) according to the manufacturer protocol^38^. The absorbance ACC deaminase was measured at λ = 540 nm. The ability to synthesize ACC deaminase was tested in the liquid shaken cultures.

### Synthesis of EPS

The ability of the tested strains to synthesize EPS was determined on LB (Biomaxima, Lublin, Poland) medium supplemented with glucose (Chempur, Piekary Śląskie, Poland) (2.5 g/L). The medium was inoculated with 5% v/v of bacterial suspension (OD = 0.1) in 0.9% NaCl (POCH, Lublin, Poland). Cultures were grown for 2, 5, and 8 days at 28 °C with 140 rpm shaking (Innova 2300 Platform shaker, New Brunswick, Edison, New Jersey, USA). After this time, the final OD600 of the cultures was determined using Infinite 200 PRO TECAN microplate spectrophotometer (Tecan, Grödig, Austria). Bacteria were separated from the post-culture liquid by centrifugation at 10,000 rpm, 15 min, 4 °C (MPW-352, MPW, Warsaw, Poland). EPS was precipitated with 96% ethanol (Linegal Chemicals, Warsaw, Poland) at 1:1 ratio for 24 h at 4 °C. EPS was separated from the supernatant by centrifugation at 10,000 rpm, 10 min, 4 °C (MPW-352, MPW, Warsaw, Poland), and dried in the Concentrator Plus Eppendorf (Eppendorf, Hamburg, Germany) for 16 h (2 × 8 h) at 45 °C V-AL and weighed. The amount of EPS obtained was converted to a volume of 1L of post-culture liquid (mg/L)^39,40^.

### Biofilm formation

96-well plates made of polystyrene (NEST, Genoplast, Rokocin, Poland) were used for biofilm formation. Strains were grown on LB (Biomaxima, Lublin, Poland) medium supplemented with glucose (Chempur, Piekary Śląskie, Poland) (2.5 g/L) at a volume of 200 µl/well. Each well was inoculated with 5% v/v of bacterial suspension (OD = 0.1) in 0.9% NaCl (POCH, Lublin, Poland). The culture was incubated for 2, 5, and 8 days at 28 °C. After this time, the medium was removed from the wells. Each well was gently rinsed with distilled water. The plate was left to air dry for a period of 25 min. After this time, 220 µl of 0.1% crystal violet (Pol Aura, Różnowo, Poland) in 1% phenol (Merck, Darmstadt, Germany) was added to each well and incubated for 15 min. After this time, excess violet was removed and each well was washed gently 3 times with distilled water. The plate was left to dry for a period of 15 min. Then 240 µl of 96% ethanol (Linegal Chemicals, Warsaw, Poland) was added to each well and shaken at 220 rpm for 15 min (Thermo-Shaker MB100-4A, µLAB, Lublin, Poland). After this time, 200 µl of the solution was transferred to a new multi-well plate and the absorbance at 560 nm was determined using Infinite 200 PRO TECAN microplate spectrophotometer (Tecan, Grödig, Austria)^41,42^. In order to determine the level of biofilm formation by the tested strains, the critical OD (ODK) was calculated according to the formula: ODK = mean OD of control + (3 × standard deviation value). Biofilm formation efficiency was ranked based on the relationship between ODK and OD of the test sample: no biofilm—OD ≤ ODK; weak biofilm—ODK < OD ≤ 2ODK; medium biofilm—2ODK ≤ OD ≤ 4ODK; strong biofilm—4ODK < OD.

### Phenotypic Profiling using BiologTM GEN III MicroPlates

Pure cultures of the rhizosphere bacteria were characterized based on a Biolog GEN III system (Biolog Inc. Hayward, CA, USA) following the manufacturer instructions. The ability to metabolize various substrates belonging to amino acids, carbohydrates and carboxylic acids, is determined through the reduction of colorless tetrazolium dye to purple formazan. In our case the analysis was applied to characterize the strains in terms of variability of utilized substrates and resistance to unfavorable conditions. It can be assumed that strains that intensively metabolize a wide range of substrates, and at the same time show resistance to high salinity, contamination or acidic pH, are better adapted to harsh conditions or a condition change.

Single bacterial colonies were transferred to inoculating fluid A (IFA) using a sterile cotton swab in order to create a suspension of bacterial cells, the transmittance of which was set in the range of 95 to 98% using a turbidimeter (BiologTM). Then, 100 µl of cell suspension was added to each well of the plate. The absorbance of each well was read at 590 nm on a Biolog MicroStation™ at 24-h intervals for 7 days.

### Identification of the bacteria

Genomic DNA was extracted from the bacterial isolates using a Bead-Beat Micro AX Gravity according to the manufacturer protocol. The following primers were used in the PCR reaction: 27F and 1492R. Afterwards, amplification and 16S rRNA Sanger sequencing was performed at the Genomed Laboratory in Warsaw, Poland. The identification of the isolates was performed using the Ribosomal Database Project (http://rdp.cme.msu.edu/) and BLAST (http://blast.ncbi.nlm.nih.gov/blast/Blast.cgi). The sequences of all the strains were submitted to the National Center for Biotechnology Information (NCBI).

The identification and GenBank accession numbers of the 15 rhizosphere bacteria are presented in Table 1. Based on 16S rRNA gene sequence similarity, the isolates were assigned to eight different genera:: *Microvirga* (1 strain), *Pseudomonas* (4 strains), *Burkholderia* (1 strain), *Rhizobium* (4 strains), *Phyllobacterium* (2 strains), *Inquilinus* (1 strains), *Mesorhizobium* (1 strain) and *Olivibacter* (1 strain). The 16S rRNA gene sequences of the isolated strains showed a high degree of similarity to the closest type strains deposited in the GenBank database, ranging from 99.70% to 100%. These strains were affiliated with two phyla: Pseudomonadota (14 strains), and Bacteroidota (1 strain). It is important to note that taxonomic classification was based solely on 16S rRNA gene sequence similarity, and therefore should be considered preliminary. Further genomic analyses will be necessary to confirm species-level identification. Phylogenetic analysis of the isolates is presented in figure S3.

**Table 1:** Identification of rhizosphere bacteria by 16S rRNA gene sequence analysis.

Number	Isolate no.	Closest known relative	16S rRNA similarity (%)	Phylum	GenBank accession no.^1^
1	LC6B	*Microvirga* sp	99.70	Pseudomonadota	PP529722
2	LC6C	*Pseudomonas* sp*.*	99.92	Pseudomonadota	PP529723
3	LC7	*Burkholderia* sp.	100	Pseudomonadota	PP529724
4	LC8	*Pseudomonas caspiana*	99.93	Pseudomonadota	PP529725
5	LC9	*Rhizobium leguminosarum*	100	Pseudomonadota	PP529726
6	LC11	*Phyllobacterium* sp*.*	100	Pseudomonadota	PP529727
7	LC12	*Rhizobium jaguaris*	100	Pseudomonadota	PP529728
8	LC13	*Rhizobium alamii*	100	Pseudomonadota	PP529729
9	LC14	*Rhizobium alamii*	100	Pseudomonadota	PP529730
10	LC16	*Phyllobacterium ifriqiyense*	100	Pseudomonadota	PP529731
11	LC17	*Olivibacter soli*	100	Bacteroidota	PP529732
12	LC18	*Mesorhizobium* sp*.*	100	Pseudomonadota	PP529733
13	LC19	*Pseudomonas* sp*.*	100	Pseudomonadota	PP529734
14	LC21	*Pseudomonas* sp*.*	99.84	Pseudomonadota	PP529735
15	LC22	*Inquilinus ginsengisoli*	99.84	Pseudomonadota	PP529736

^1^Unique identifier in the genetic sequence database: https://www.ncbi.nlm.nih.gov/genbank/. The date of sequence deposition in GenBank: March 30, 2024.

### 2.10. Greenhouse pot experiment

#### Experimental set up

The pot study was set up in 2022 in a greenhouse to evaluate the effects of soil inoculation with three selected isolates (LC7—*Burkholderia sp*.; LC8—*Pseudomonas caspiana*; LC11—*Phyllobacterium sp*.). The strains for the pot study were selected to represent various species characterized by regrowth after freeze-drying and presence of plant growth promotion mechanisms important in stress conditions: ACC and biofilm production. Additional criteria taken into account were other useful mechanisms from the perspective of the potential use of bacteria: phosphorus solubilization potential, nitrogen fixation and/or IAA production.

The experiment was carried out under controlled conditions (supplemental light and 27/20 C day/night temperatures) in 3 L plastic pots with three replicates for each combination at 2 soil moisture levels. The soil had a texture of sandy loam, containing 0.69% of organic carbon and characterized by neutral pH (6.5 in water slurry). The soil had an average level of available phosphorus (12.3 mg/100 g) according to the Egner-Riehm method. Two levels of moisture regime were set-up, including optimal conditions (kept at 60% of field water holding capacity, FWHC), and the deficit soil moisture (kept at 40% of FWHC) over the entire experiment.

Wheat was selected as the test plant as a crop with great importance both in Poland and globally. Moreover, it is a drought-sensitive crop, significantly affected by insufficient precipitation in the vegetation period. The pot test was run with three replicates for each combination.

Each pot was inoculated with the bacterial strains suspended in 10 mM MgSO_4_ (homogeneous cell density—10^7^ CFU/ml) during the germination period (approximately 3 weeks after sowing) with 100 mL of each bacterial suspension. The same amount of sterile 10 mM MgSO_4_ was added to the non-inoculated pots. Inoculation was repeated 3 weeks later, using the same procedure.

For bacterial inoculation, fresh cultures of bacterial strains were grown in liquid media. Cultures were set up for 24 h and incubated at 28 °C in a New Brunswick Scientific incubator (Excella E24 Incubator Shaker Series). Five millilitres of this pre-culture was then transferred to fresh liquid media, respective for a given strain, and incubated for 12 h at 28 °C. Subsequently, the bacterial biomass was harvested by centrifugation (6000 rpm, 15 min), washed once with sterile 10 mM MgSO_4_ and re-suspended in 10 mM MgSO_4_ to an OD660 of 1.0 (about 10^7^ cells per mL)^9^.

Each pot was inoculated with the bacterial strains during the germination period (approximately 3 weeks after sowing) with 100 mL of each bacterial suspension. The same amount of sterile 10 mM MgSO_4_ was added to the non-inoculated pots. Inoculation was repeated 3 weeks later, using the same procedure.

The experiment lasted for 3 months, after this time the plants were harvested, dried, and the roots and aboveground parts weighed. Soil samples were collected for analysis of soil enzyme activities and selected chemical parameters.

#### Post-experiment sample analyses

Moist soil samples were subjected to determination of the activities of acid (AcP) and alkaline phosphatase (AlP), measured with PNP (sodium p-nitrophenylphosphate), following the colorimetric method described by Tabatabai and Bremner^43^. Soil dehydrogenases activity (DHA) was determined by measuring the reduction of TTC (triphenyltetrazolium chloride) to triphenylformazan (TPF) according to the method of Casida et al.^44^. Each measurement was performed in triplicate.

### Statistical analysis

All the data are presented as means ± standard deviation (SD). The statistical analysis was performed using Statistica v. 13.0 (TIBCO Software Inc., Palo Alto, CA, USA). The data were subjected to analysis of variance (ANOVA). The significance of difference was identified using Scheffé test. ANOVA revealed significant variability for all the measured microbiological indices at p > 0.05. Relationships between the microbiological parameters were evaluated using Spearman correlation test. Principal component analysis (PCA) was also applied to explain the relationships between the studied microbiological parameters and their similarities. Prior to the PCA, the data were centered, and log transformed. UPGMA as a simple agglomerative hierarchical clustering method was used for the additional analysis of similarities between the strains. The data from Biolog GEN III experiments were combined in a single matrix for presentation as a heat map graph. The Biolog GEN III system provides functional diversity indices, such as average well color density (AWCD), Shannon diversity index (H), Evenness index (E), and Simpson’s diversity index (D) were calculated for data after 168 h. The data from the the pot study was subjected to two-way ANOVA. Since the interaction effects between soil moisture and strains were significant, the means are presented within a moisture regime. The significance of difference between the strain effects within a given soil moisture regime was identified using Tukey’s test (p < 0.05).

## Results

### Phosphate solubilization index

All bacterial isolates were able to solubilize tricalcium phosphate in solid media, exhibited by formation of the halo zones around bacterial colonies (Table 2). According to the Berraquero et al. classification, two separate groups were created, i.e. the isolates were grouped as medium (2 < SI ≤ 4), or high solubilization efficiency (SI > 4)^31^, however only LC18—*Mesorhizobium sp*.was classified as highly solubilising strain (SI 4.02). This strain solubilized significantly more P than most of other bacteria. All other bacterial isolates were classified as solubilizers with medium degree of solubilization, however, four of them exceeded value 3: LC6C—*Pseudomonas sp.*, LC11- *Phyllobacterium sp*., LC13—*Rhizobium alamii*, LC16—*Phyllobacterium ifriqiyense*.

**Table 2:** Production of indole-3-acetic acid (IAA)-like compounds and phosphate solubilisation activity by the tested strains. The values presented are means and standard deviations (*SD*; n = 3).

Isolate no	Strain	IAA-like compounds production (µg/mL)	Phosphate solubilization	
			index of PSB (SI)	Zone size^1^
LC6B	*Microvirga* sp	10.39^bcd2^ ± 2.91	2.66^bc^ ± 0.15	middle
LC6C	*Pseudomonas* sp*.*	0.00^d^ ± 0.00	3.28^abc^ ± 0.25	middle
LC7	*Burkholderia* sp.	1.35^d^ ± 0.11	2.33^bc^ ± 0.17	middle
LC8	*Pseudomonas caspiana*	12.24^bcd^ ± 1.30	2.23^c^ ± 0.06	middle
LC9	*Rhizobium leguminosarum*	14.82^bcd^ ± 1.08	2.43^bc^ ± 0.13	middle
LC11	*Phyllobacterium* sp*.*	24.87^b^ ± 3.67	3.16^abc^ ± 0.55	middle
LC12	*Rhizobium jaguaris*	25.01^b^ ± 7.53	2.31^bc^ ± 0.17	middle
LC13	*Rhizobium alamii*	6.80^ cd^ ± 0.62	3.31^ab^ ± 0.30	middle
LC14	*Rhizobium alamii*	2.90^d^ ± 0.14	2.26^bc^ ± 0.21	middle
LC16	*Phyllobacterium ifriqiyense*	3.92^ cd^ ± 1.31	3.27^abc^ ± 0.15	middle
LC17	*Olivibacter soli*	8.99^ cd^ ± 1.20	2.56^bc^ ± 0.20	middle
LC18	*Mesorhizobium* sp*.*	60.11^a^ ± 0.02	4.02^a^ ± 0.73	high
LC19	*Pseudomonas* sp*.*	18.32^bc^ ± 0.98	2.78^bc^ ± 0.29	middle
LC21	*Pseudomonas* sp*.*	11.11^bcd^ ± 0.50	2.27^bc^ ± 0.13	middle
LC22	*Inquilinus ginsengisoli*	10.67^bcd^ ± 0.86	2.24^c^ ± 0.13	middle

^1^The isolates were grouped as middle (2 < SI ≤ 4), and high solubilization efficiency (SI > 4), according to publication of Berraquero et al. 1976.

^2^Means marked with the same letter do not differ significantly (*p* < 0.05, n = 3) according to the Scheffé test.

### Potential for nitrogen-fixing

The efficiency of N_2_ binding by the tested strains was checked based on the increase in total N content in bacterial cultures in liquid nitrogen-free medium after 24 h, 48 h and 72 h of incubation. All tested strains fixed N to some extent (Table 3). The most effective N assimilator was: LC8—*Pseudomonas caspiana* (341.9 mg of nitrogen/ml after 72), while LC7—*Burkholderia sp*. exhibited the lowest effectiveness (8.85 mg of nitrogen/ml). The rest of bacterial isolates showed the ability to fix atmospheric nitrogen in the range from 15.85 to 50.00 mg/ml after 72 h.

**Table 3:** Nitrogen-fixing activity of the tested bacterial strains (mg/ml). The values are means with standard deviation (*SD*; n = 3).

Isolate no	Strain	24 h	48 h	72 h
LC6B	*Microvirga* sp	25.13^e1^ ± 0.93	21.99^cdef^ ± 0.10	27.40^ cd^ ± 0.23
LC6C	*Pseudomonas* sp*.*	40.03^b^ ± 0.66	41.60^b^ ± 0.38	50.00^b^ ± 1.28
LC7	*Burkholderia* sp.	6.68^i^ ± 0.57	7.17^e^ ± 0.43	8.85f. ± 0.18
LC8	*Pseudomonas caspiana*	83.07^a^ ± 2.07	276.53^a^ ± 16.56	341.90^a^ ± 9.80
LC9	*Rhizobium leguminosarum*	12.56^ h^ ± 0.19	14.02^ef^ ± 0.59	16.65^def^ ± 0.36
LC11	*Phyllobacterium* sp*.*	36.52^c^ ± 0.27	38.02^bcd^ ± 0.09	36.85^c^ ± 0.77
LC12	*Rhizobium jaguaris*	19.10^ fg^ ± 0.48	25.81^bcdef^ ± 0.95	28.93^c^ ± 0.44
LC13	*Rhizobium alamii*	21.88^efg^ ± 0.52	22.72^bcdef^ ± 0.75	26.52^cde^ ± 0.19
LC14	*Rhizobium alamii*	12.86^ h^ ± 0.52	14.67^ef^ ± 0.70	17.32^def^ ± 0.22
LC16	*Phyllobacterium ifriqiyense*	13.22^ h^ ± 0.35	13.20^ef^ ± 0.21	15.85^ef^ ± 0.41
LC17	*Olivibacter soli*	29.42^d^ ± 0.44	31.07^bcde^ ± 0.48	29.33^c^ ± 1.37
LC18	*Mesorhizobium* sp*.*	39.49^bc^ ± 0.93	39.71^bc^ ± 1.82	36.12^c^ ± 0.30
LC19	*Pseudomonas* sp*.*	22.30^ef^ ± 0.28	22.99^bcdef^ ± 0.58	27.03^cde^ ± 0.10
LC21	*Pseudomonas* sp*.*	24.08^e^ ± 0.54	20.47^def^ ± 0.37	30.25^c^ ± 0.81
LC22	*Inquilinus ginsengisoli*	26.25^ g^ ± 1.58	28.33^bcde^ ± 0.47	30.93^c^ ± 0.44

^1^Means marked with the same letter do not differ significantly (*p* < 0.05, n = 3) according to the Scheffé test.

### Potential for IAA-like compounds production

The assay showed that bacterial isolates, except LC6C—*Pseudomonas sp*., were able to produce IAA on TSB medium with L-Trp at variable levels (Table 2). The highest IAA concentration was found in the culture filtrate of LC18—*Mesorhizobium sp*. (60.11 μg/ml), whereas, the isolate LC7 showed minimum IAA production.

### Potential ACC deaminase activity

**Fig. 1:**
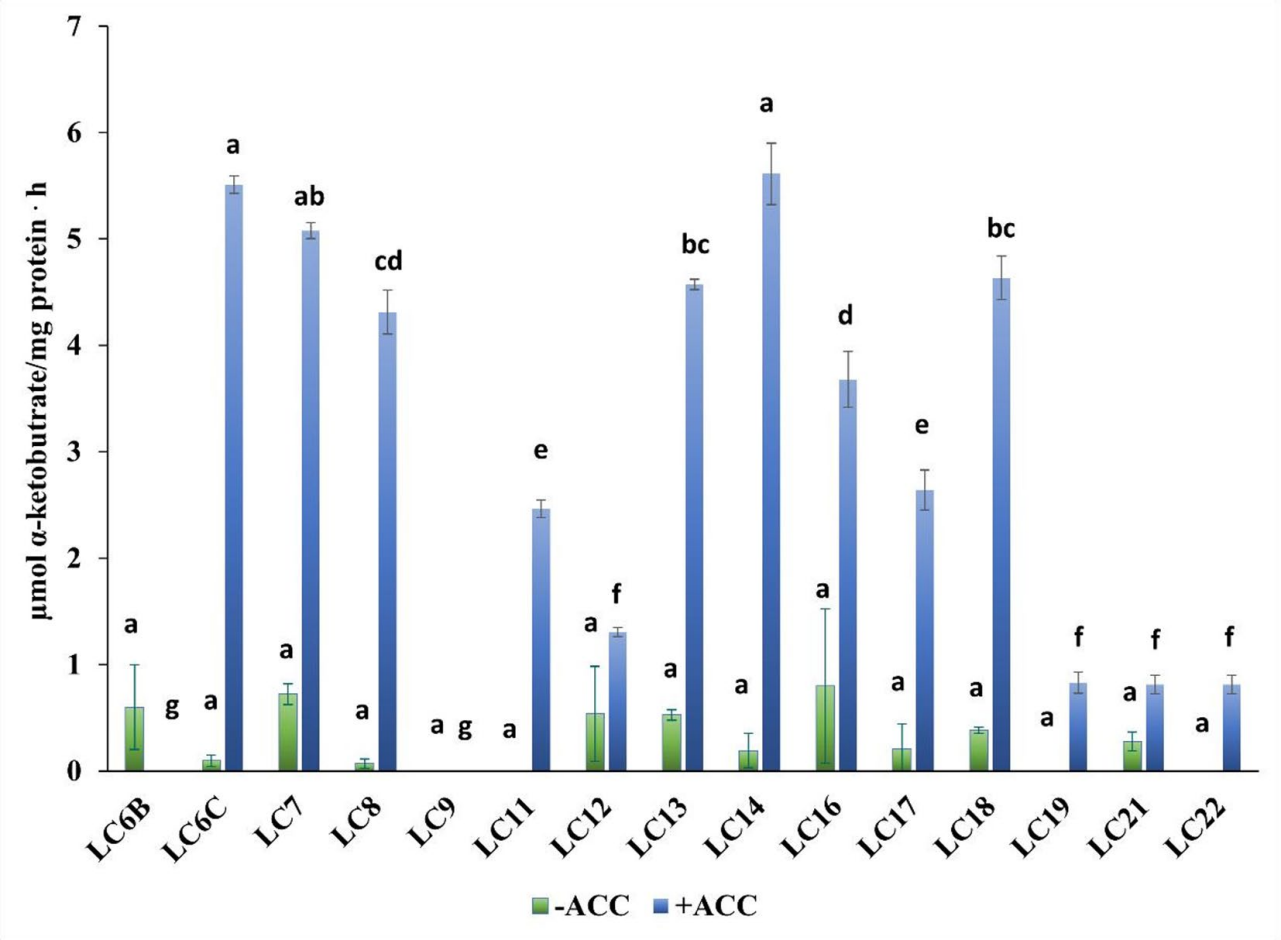
ACC deaminase activity values of the rhizosphere bacteria after 72 h with and without ACC in the medium. Bars indicate standard deviation (*SD*). Means marked with the same letter do not differ significantly (*p* < 0.05, n = 3) across the treatment, according to the Scheffé test.

Fourteen strains of the 15 tested exhibited ACC deaminase activity (Fig. 1). The highest activity was observed for LC14—*Rhizobium alamii* (5.61 µmol α-KB/mg protein h), followed by LC6C—*Pseudomonas sp*. (5.51), and LC7—*Burkholderia sp*. (5.07). The lowest ACC deaminase production was observed for LC19—*Pseudomonas sp*. (0.83); LC21—*Pseudomonas sp*. (0.81); LC22—*Inquilinus ginsengisoli* (0.81) (Fig. 1). In the medium without ACC, the ability to synthesize ACC deaminase was demonstrated only for 11 strains. Among these isolates, the strain LC16—*Phyllobacterium ifriqiyense* (0.81 µmol/mg protein h) and LC7—*Burkholderia sp*. (0.81) showed the greatest activity. The addition of ACC as a N source significantly increased the efficiency of ACC deaminase biosynthesis. Strain LC9—*Rhizobium leguminosarum* did not show the ability to produce deaminase.

### Production of EPS

**Fig. 2:**
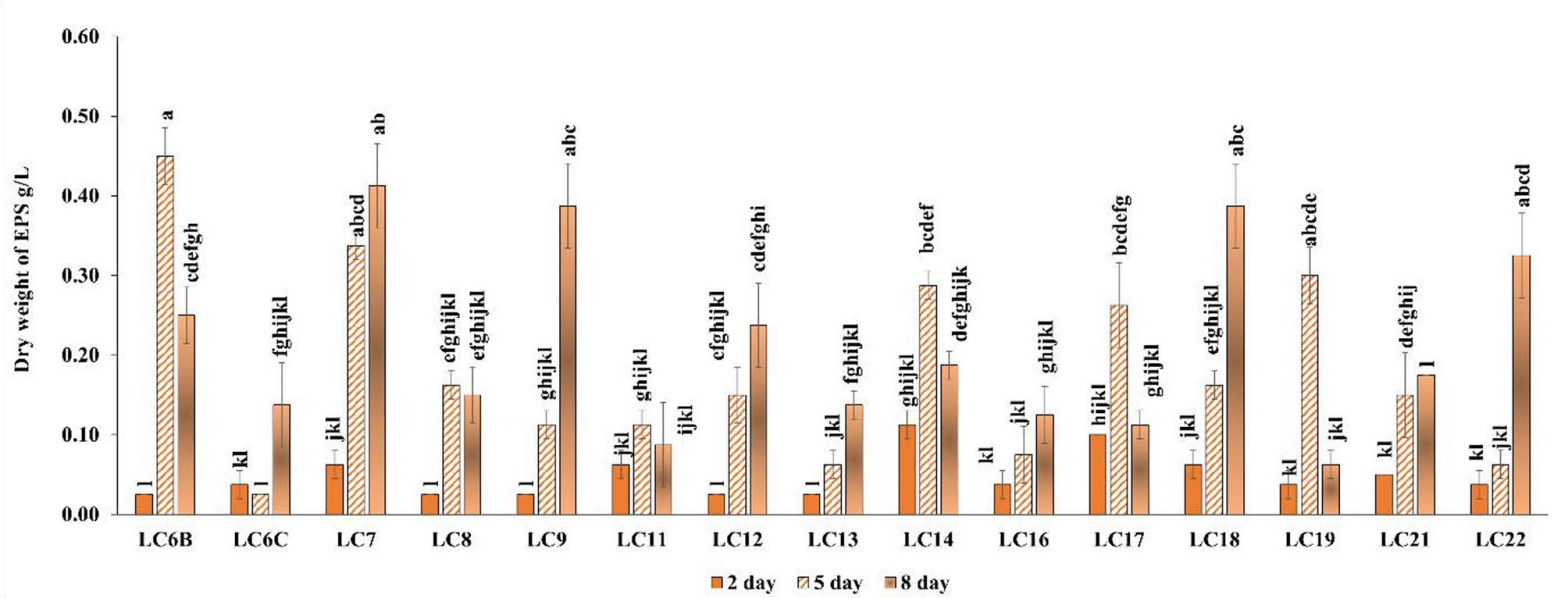
Dynamics of EPS synthesis by the tested bacterial strains during the culture growth for 2, 5 and 8 days. Bars indicate standard deviation (*SD*). Means marked with the same letter do not differ significantly (*p* < 0.05, n = 3) across time of measurement, according to the Scheffé test.

All the strains tested showed the ability to synthesise EPS. The highest EPS synthesis was observed for LC6B—*Microvirga sp*. on day 5 of the incubation—at 0.45 g/L, significantkly greater than in case of other strains on that day, followed by a decrease in the amount of EPS after 8 days (Fig. 2). Strains LC7—*Burkholderia sp*., LC9—*Rhizobium leguminosarum* and LC18—*Mesorhizobium sp*. synthesised EPS at a slightly lower level (approx. 0.4 g/L), but only after 8 days of culture growth. In contrast to LC6B—*Microvirga sp*., these strains showed an increase in EPS synthesis with the incubation time.

### Formation of biofilm

**Fig. 3:**
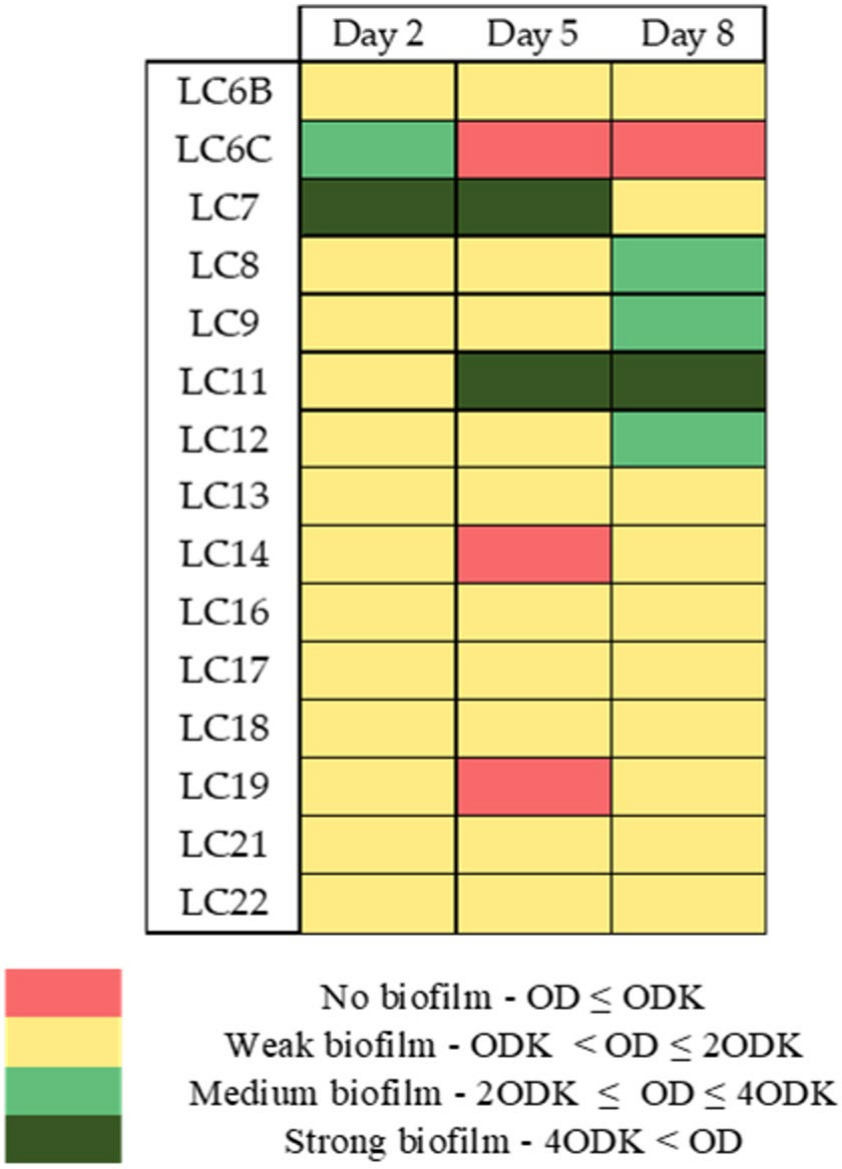
Production of biofilm by bacteria strains during the culture growth period of 2, 5, 8 days.

The ability of the tested strains to produce biofilms was highly diverse and dependent on the day of observation. Most of the tested isolates synthesized biofilms at very low levels (ODK < OD ≤ 2ODK). The highest level of biofilm formation (4ODK < OD) was observed for strain LC7—*Burkholderia sp*. after 2 and 5 days and for strain LC11—*Phyllobacterium sp*. after 5 and 8 days of incubation (Fig. 3). On the second day of culture, the LC6C—*Pseudomonas sp*. synthesized biofilm at medium level (2ODK < OD ≤ 2ODK) but did not produce substantial biofilms in the following days, while LC8—*Pseudomonas caspiana*; LC9—*Rhizobium leguminosarum* and LC12—*Rhizobium jaguaris* intensified the biofilm production to medium level after 8 days (Fig. 3).

### Phenotypic profiling of bacteria

The analysis based on AWCD revealed the diverse metabolic patterns (metabolic fingerprint) of the tested bacteria. GEN III MicroPlatesTM testing involved 69 types of carbon sources, including amino acids (10 types), carbohydrates (28 types), carboxylic acids (22 types) and selected sensitivity tests (9 types) (Figure S4). The highest utilisation of amino acids was observed for the strains LC6C—*Pseudomonas sp*., LC7—*Burkholderia sp*., LC8—*Pseudomonas caspiana* and LC21—*Pseudomonas sp*. Among the strains that most actively metabolized carbohydrates, we distinguished: LC13—*Rhizobium alamii*, LC14—*Rhizobium alamii*, LC16—*Phyllobacterium ifriqiyense*, LC17—*Olivibacter soli* (Figure S4). Among carbohydrates, the lowest substrate utilization was observed for 3-Methyl Glucose and D-arabitol. The list of bacteria that intensively utilized carboxylic acids included: LC6C—*Pseudomonas sp*.; LC7—*Burkholderia sp*.; LC8—*Pseudomonas caspiana*; LC19—*Pseudomonas sp*.; LC21—*Pseudomonas sp*. Considering individual carbon sources, it was observed that acetoacetic acid was the least metabolized by all the bacteria (Figure S4).

Chemical sensitivity tests revealed large variability responses across the strains tested. Regardless of the pH value, we could observe that the greatest activity was demonstrated by the strains LC6C—*Pseudomonas sp*., LC7—*Burkholderia sp*., LC8—*Pseudomonas caspiana* and LC21—*Pseudomonas sp.* Metabolic activity of these strains is not affected by the mild pH shift. At high salinity conditions (4 and 8% NaCl) the strains did not show the ability to metabolize the substrates, whereas at mild salinity (1% NaCl) some strains were active. Lithium chloride was suppresive to practically all strains (Figure S4). Based on the obtained heatmaps, it can be concluded that the metabolic activity of the strains towards the sensitivity tests, was quite low (Figure S4).

Phenotypic microarrays allowed the identification of differences in profiles between the tested species. The analysis showed low activity for the LC12 bacteria—*Rhizobium jaguaris* to metabolize all compounds. IN contrast, high metabolic activity was demonstrated for the bacteria LC21—*Pseudomonas sp*. across various carbon sources (Figure S4).

The GEN III MicroPlatesTM results were used to calculate AWCD and diversity indexes across carbon sources. The C utilization profiles revealed a broad variability between individual bacteria strains (Table 4). The highest metabolic activity expressed by AWCD was demonstrated for the LC21 strain of *Pseudomonas sp.* (AWCD = 136.83). Opposingly, the strain LC12—*Rhizobium jaguaris* (AWCD = 15.03) was characterized by the lowest rate of C use. The H, E and D indexes, representing diversity of metabolic activities of the tested bacteria, are presented in Table 4. The H ranged from 4.46 (LC9—*Rhizobium leguminosarum*) to 3.69 (LC12—*Rhizobium jaguaris*), which indicates high metabolic diversity for most of bacteria tested in the current study. Based on H and D values, the strain LC9—*Rhizobium leguminosarum* was characterized by the highest diversity indexes of the utilized substrates, but lower rate of the utilization. Among most active strains, LC17—Olivibacter soli exhibited high diversity of C sources.

**Table 4:** Indicators characterizing activity of the tested strains in the carbon utilization and diversity of carbon sources. Means and standard deviation (*SD*; n = 3) are presented.

Isolate no	Strain	AWCD	Shannon diversity index (H)	Evenness index(E)	Simpson diversity index (D)
LC6B	*Microvirga* sp	26.37^d1^ ± 6.06	3.88^de^ ± 0.14	0.88^d^ ± 0.02	0.97^c^ ± 0.00
LC6C	*Pseudomonas* sp*.*	133.87^ab^ ± 3.60	4.18^abcd^ ± 0.01	0.93^abcd^ ± 0.00	0.98^ab^ ± 0.00
LC7	*Burkholderia* sp.	118.27 ^ab^ ± 5.25	4.17 ^abcd^ ± 0.01	0.95^abc^ ± 0.00	0.98^ab^ ± 0.00
LC8	*Pseudomonas caspiana*	124.93 ^ab^ ± 5.36	4.16 ^abcd^ ± 0.02	0.93^bcd^ ± 0.00	0.98^ab^ ± 0.00
LC9	*Rhizobium leguminosarum*	41.00^d^ ± 8.82	4.46^a^ ± 0.03	0.97^a^ ± 0.00	0.98^a^ ± 0.00
LC11	*Phyllobacterium* sp*.*	56.05^ cd^ ± 7.03	3.95^cde^ ± 0.15	0.89^ cd^ ± 0.00	0.97^bc^ ± 0.00
LC12	*Rhizobium jaguaris*	15.03^d^ ± 2.48	3.69^e^ ± 0.10	0.85^e^ ± 0.01	0.96^d^ ± 0.00
LC13	*Rhizobium alamii*	113.15 ^ab^ ± 5.40	4.16 ^abcd^ ± 0.03	0.92^bcd^ ± 0.00	0.98^ab^ ± 0.00
LC14	*Rhizobium alamii*	88.25^bc^ ± 5.90	4.00^bcd^ ± 0.07	0.90^bcd^ ± 0.01	0.97^abc^ ± 0.00
LC16	*Phyllobacterium ifriqiyense*	97.45^abc^ ± 12.44	4.05^bcd^ ± 0.10	0.91^abcd^ ± 0.00	0.98^abc^ ± 0.00
LC17	*Olivibacter soli*	130.77 ^ab^ ± 26.68	4.29^abc^ ± 0.13	0.94^abc^ ± 0.00	0.98^ab^ ± 0.00
LC18	*Mesorhizobium* sp*.*	95.71 ^abc^ ± 11.82	4.18^abcd^ ± 0.05	0.94^abc^ ± 0.00	0.98^ab^ ± 0.00
LC19	*Pseudomonas* sp*.*	103.60 ^ab^ ± 16.23	4.15 ^abcd^ ± 0.09	0.91^ cd^ ± 0.01	0.98^abc^ ± 0.00
LC21	*Pseudomonas* sp*.*	136.83^a^ ± 8.85	4.21^abc^ ± 0.03	0.94^abc^ ± 0.00	0.98^ab^ ± 0.00
LC22	*Inquilinus ginsengisoli*	40.43^d^ ± 6.11	4.41^ab^ ± 0.04	0.96^ab^ ± 0.01	0.98^ab^ ± 0.00

^1^Means marked with the same letter do not differ significantly (*p* < 0.05, n = 3) according to the Scheffé test.

### Principal component analysis and correlation between microbiological parameters

The correlation analysis revealed positive relationship between AWCD and ACC deaminase activity whereas AWCD was negatively correlated to biofilm formation and EPS production (Table S2). Strains intensively fixing N in general released IAA quite intensively but not necessarily were active in producing EPS. IAA production levels were negatively correlated with ACC and AWCD.

**Fig. 4:**
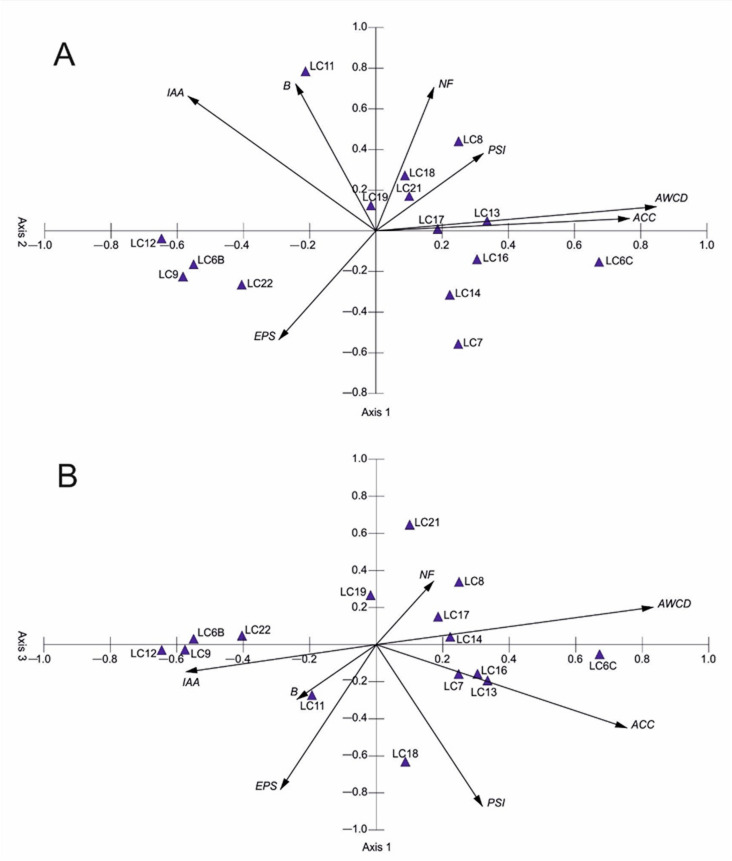
Principal component analysis (PCA) for microbiological parameters measured for 15 bacterial strains (**A**—axis 1 and 2 ordination; **B**—axis 1 and 3 ordination).

Figure 4 shows the results of the PCA ordination of the measured microbial characteristics. The eigenvalues of the first (2.16), second (1.53), and third axes (1.20) indicate the presence of three gradients, within all strains differentiated in terms of the analyzed properties. The first three axes explained 69.84% of the variability. On the right side of the ordination space we can observe strains with the highest values of ACC and AWCD, and less intensively producing IAA and EPS, while on the left side strains exhibiting lower ACC and AWCD, and higher IAA and EPS. The peripheral part of the ordination space is occupied by LC11, which has the highest biofilm production and one of the highest IAA and phosphate solubilization index. Centrally located LC19 is characterized by medium values of majority of analyzed strain characteristics (Fig. 4).

The cluster analysis confirmed similarities between the strains highlighted by the PCA. Clustering showed that two separate groups: the group consisting of LC8, LC16, LC14, LC17, LC13, LC7, LC6C, and the group consisting of LC21, LC19, LC18, LC11,LC12, LC22, LC9, LC6B, are clearly distinguished (Figure S5). The percent similarity of the two groups is 85.2%. Similarity over 95% is characteristic for the following pairs of strains: LC16 and LC14, LC17 and LC13, LC22 and LC9.

### The pot experiment results

The pot study enabled preliminary testing of three strains, selected based on the laboratory measurements, towards wheat growth promotion. *Burkholderia sp.* was characterized by intensive biofilm and EPS production, *Phyllobacterium sp.* as IAA and biofilm producer, and P solubilizer, *Pseudomonas caspiana* effective in solubilizing P and fixing N.

The analysis of plant biomass results revealed that soil inoculation with all the tested isolates stimulated plant growth at both soil moisture levels (Table 5). However this effect was more pronounced under the moisture deficiency. At optimal moisture the strains applied significantly increased shoot and total biomass. The application of the strains to the low moisture soil led to the sharp enhancement of root development of wheat, that very likely helped to increase also the shoot biomass. It is important to mention that after addition of LC7, LC8 and LC11 to the low moisture soil, the shoot biomass was not lower than the biomass produced under optimal conditions (Table 5).

**Table 5:** Plant biomass as dependent on the soil inoculation and the moisture level.

Moisture	Inoculation	Shoot biomass [g]	Root biomass [g]	Total biomass [g]
Optimal	Control	1.32 ± 0.09^c^	0.67 ± 0.25^a^	1.99 ± 0.23^b^
Optimal	LC7	1.63 ± 0.06^ab^	0.97 ± 0.13^a^	2.60 ± 0.16^a^
Optimal	LC8	1.55 ± 0.05^b^	1.03 ± 0.28^a^	2.58 ± 0.10^a^
Optimal	LC11	1.68 ± 0.06^a^	0.91 ± 0.05^a^	2.59 ± 0.18^a^
Low	Control	1.31 ± 0.02^b^	0.54 ± 0.19^b^	1.85 ± 0.15^c^
Low	LC7	1.57 ± 0.04^a^	1.75 ± 0.17^a^	3.32 ± 0.13^a^
Low	LC8	1.48 ± 0.07^a^	1.43 ± 0.28^a^	2.91 ± 0.21^b^
Low	LC11	1.51 ± 0.07^a^	1.83 ± 0.25^a^	3.34 ± 0.26^ab^

*Means marked with the same letter did not differ significantly across the inoculation treatments within the soil moisture level (*p* < 0.05, n = 3) according to the Tukey test.

Phosphatases activity measured after plant harvest was not reduced due to inoculation. In some cases inoculated soils exhibited greater activity of acid (LC8, optimal moisture; LC7, low moisture) and alkaline phosphatases (LC7, optimal moisture; LC8, low moisture). Dehydrogenase activity was enhanced after addition of tested strains (Table 6).

**Table 6:** Enzymatic activity of soil after wheat harvest as dependent on the soil inoculation and the moisture level.

Moisture	Inoculation	Acid phosphatase activity	Alkaline phosphatase activity	Dehydrogenase activity
		[µg PNP/g dm h]	[µg PNP/g dm h]	[µg TTC/ g dm h]
Optimal	Control	17.48 ± 0.76^b^	40,88 ± 3.50^b^	50.49 ± 6.06^c^
Optimal	LC7	18.08 ± 0.55^b^	50.22 ± 2.24^a^	61.39 ± 6.43^bc^
Optimal	LC8	19.26 ± 0.31^a^	43.10 ± 4.66^b^	69.83 ± 2.40^b^
Optimal	LC11	17.96 ± 0.46^b^	42.82 ± 0.73^b^	82.22 ± 5.41^a^
Low	Control	11.48 ± 1.97^b^	35,17 ± 3.53^b^	44.65 ± 2.49^b^
Low	LC7	15.39 ± 0.56^a^	37,30 ± 2.47^ab^	62.98 ± 2.74^a^
Low	LC8	14.03 ± 1.88^ab^	42,92 ± 3.91^a^	59.43 ± 3.91^a^
Low	LC11	15.59 ± 1.75^ab^	36,80 ± 2.40^b^	65.83 ± 3.57^a^

Means marked with the same letter did not differ significantly across the inoculation treatments within the soil moisture level (p < 0.05, n = 3) according to the Tukey test.

## Discussion

The interest in the use of rhizospheric bacteria as a method to improve crop production is constantly increasing^45^. The key stages for developing an effective biofertilizer include: selection of appropriate plants and root zone soil for the isolation of strains, isolation of bacteria, cultivation of isolates in laboratory conditions, examination of key mechanisms biostimulating plant growth, strain identification and testing strain effectiveness in plant growth promotion^46^. The most effective strains in laboratory and greenhouse conditions can be used for further field testing, and after confirming their effectiveness in real conditions, become effective components of biofertilizers.

Our study, covering a range of plant growth promotion characteristics, revealed that all the tested bacterial strains from the extreme environment have certain biotechnological potential to improve plant growth. However, from the practical point of view, the crucial strain characteristic is its ability to regrow after strain freeze-drying and storage. Our tests proved that 13 of 15 strains were able to quickly regrow after freeze-drying. This test constituted the first step in the strain selection. Lack of ability to regrow after freeze-drying limits the potential of using these bacteria in biofertilisers only to some extent since alternative methods to preserve their viability can be used, such as encapsulation, spray drying or vacuum drying.

The isolates belonged to Pseudomonadota and Bacteroidota. All of them were able to grow on phosphate substrate which indicates certain capability to dissolve phosphates also in soil. Literature data confirm that the use of PSB as inoculants contributes to increased P uptake by plants and an increase in yields^47^. There is also a great diversity within PSB, and among this group other authors have distinguished such genera as *Azospirillum, Bacillus, Pseudomonas, Nitrosomonas, Erwinia, Serratia, Rhizobium, Xanthomonas, Enterobacter* and *Pantoea*^48,49^. In addition to P solubilisation, these microorganisms often promote plant growth and development by: producing or changing the concentration of plant hormones such as indole acetic acid^50,51^, fixing atmospheric nitrogen^52^, synthetizing ACC deaminase, which hydrolyzes ACC to NH_3_ and α-ketobutyrate, which consequently reduces the inhibitory effect of ethylene on plant growth^53^. Such multifunctional strains seem to be potentially most promissing components of biofertilisers in sustainable agriculture and for adapatation to climate change. It is worth to screen for such strains across various types of environments, also to look for bacteria resistant to unfavorable conditions.

Despite high total contents of phosphorus in soils, its accessibility is limited since majority of phosphorus in soils exists in insoluble forms^54^. Vargas et al. has found that only less than 5% of soil phosphorus is available for uptake by plants. Therefore, one of the most important plant growth-promoting processes is ability of bacteria to convert phosphates from insoluble to soluble forms^55^. Microbes considered as the most efficient phosphate solubilizers are *Rhizobium leguminosarum, Rhizobium meliloti, Mesorhizobium mediterraneum, Bradyrhizobium sp., B. japonicum, Bacillus, Pseudomonas* and *Azotobacter*^56–58^. In the present study, all isolates were characterized by some ability to solubilize phosphates evaluated as medium ability, while LC18—*Mesorhizobium sp*. was the most effective. In another study, five bacterial isolates formed clear halos around colonies on PVK plates supplemented with calcium phosphate. They belonged to *Bacillus* and *Pseudomonas* genera. PSM are currently considered as an effective way to supply crops with phosphorus. Inoculation of soil or seeds with these microorganisms is a method to improve the solubilization of soil-bound and applied phosphate, which can thereby increase crop yields and enhance phosphorus use efficiency^29^.

Using biological methods of N fixation could be an attractive alternative to intensive use of chemical fertilizers^57^. Biological Nitrogen Fixation (BNF) is accomplished by free-living microorganisms, such as *Acetobacter spp., Azospirillum spp., Azotobacter spp., Bacillus spp., Citrobacter spp., Clostridium spp., Enterobacter spp., Klebsiella spp., Pseudomonas spp., Serratia spp.,* or *Streptomyces spp.*, or it can also be accomplished by some cereal-associated microorganisms, e.g., *Azospirillum spp., Herbaspirillum spp., and Azoarcus spp*., or rhizobia in interaction with legumes^59^. The ability to grow in nitrogen-free medium had endophytic isolates belonging to the genera *Enterobacter, Rahnella, Rhodanobacter, Pseudomonas, Stenotrophomonas, Xanthomonas* and *Phyllobacterium*^60–62^. In some studies the most effective atmospheric nitrogen assimilators were *Delftia acidovorans* and *Achromobacter xylosoxidans*^16^. In our study all strains isolated from the industrial site were able to fix air N, however *Pseudomonas caspiana* was most effective. It is assumed that nitrogen was rather deficient in the topsoil of the reclaimed smelter wasteland from where the strains were isolated, since the field was never fertilized after the reclamation. The bacteria might have evolved into greater ability to mineralize or fix N.

One of major plant growth promotion traits is IAA production. It is a fundamental phytohormone that modulates plant growth^63,64^. Bacterial IAA may promote plant growth through affecting processes such as cell division, elongation, movement reaction of plants, apical dominance and processes of senescence, flowering, and response to stress^65–67^. This phytohormone helps to produce longer roots and increases number of root hairs and lateral roots which are involved in nutrient uptake^68^. It is well known that PGPR play major role in the enhancement of the plant physiology, differentiation, expansion, and cell division by their ability to produce auxins, particularly IAA. Over 80% of rhizosphere bacteria, such as *Azospirillum, Pseudomonas, Klebsiella, Rhizobium, Mesorhizobium, Bradyrhizobium, Paenibacillus, Bacillus, Azotobacter, Enterobacter* and *Staphylococcus* actively produce and release auxins^56,69,70^. In other studies strains classified as *Delftia* genus showed IAA production above 10 μg IAA/ml^16^. Among our strains, the isolates LC6B—*Microvirga sp*.; LC8—*Pseudomonas caspiana*; LC9—*Rhizobium leguminosarum*; LC11—*Phyllobacterium sp*.; LC12—*Rhizobium jaguaris*; LC18—*Mesorhizobium sp*.; LC19—*Pseudomonas sp*.; LC21—*Pseudomonas sp*.; LC22—*Inquilinus ginsengisoli* exceded this level of IAA production. Auxins act through signal transduction mechanisms, so their role can be either positive or detrimental factor for plant growth, depending on their concentration. It should be noted that excessive exogenous auxin causes disturbances in proper functioning, including damage to the hormonal loop regulation system, and may increase the concentration of ethylene in the roots^71^. Thus, this feature may be dangerous for plants under certain conditions^72^. Therefore, literature data indicate that the final effect of auxins depends on the balance of their contents.

ACC deaminase is another key trait exhibited by PGPR. This ability is one of the key growth-stimulating features affecting stress tolerance in plants^73^. ACC deaminase-production decreases levels of plant ethylene which, when present in high concentrations, can inhibit root elongation and growth^74,75^. This enzyme is responsible for the cleavage of the plant ethylene precursor ACC into ammonia and -ketobutyrate. The presence of microbial producers of ACC deaminase in biofertilisers is important in agricultural systems introducing nature based solutions. As reported by Singh and Jha under stress conditions the ACC deaminase production of > 20 nmol of α-KB/mg protein per 1 h is sufficient to trigger systemic tolerance^74^. In our study there was a range of strains producing this enzyme at greater levels.

Bacterial biofilms are surface attached communities of bacteria connected by self-produced substances including polysaccharides, proteins and extracellular DNA. Biofilms have beneficial roles in plant protection, bioremediation or wastewater treatment^76^. EPSs contribute to structural integrity of biofilms, therefore they provide protection of microbial communities from the harsh environments. They also positively affect soil structure, improving water holding capacity of soil. Therefore strains effective in producing biofilms and EPS might be valuable components of biofertilisers dedicated to harsh conditions, such as contaminated soil or agricultural soil under frequent drought since they would be capable to survive in these conditions. Among the strains tested LC7—*Burkholderia sp*. seems to be promising since it produces biofilm and intensively produces EPS that stabilizes biofilms.

GEN III microplates enabled to analyze the ability of strains to metabolize diverse sources of carbon and determine other important physiological properties such as tolerance to pH shift, salinity, and some toxic compounds that might reduce activity of bacteria^77^. In general, bacteria classified as *Pseudomonas* were the most metabolically active when expressed as AWCD index, even if the highest diversity indexes were attributed to other strains. Moreover, all *Pseudomonas* strains genus were characterized by metabolic activity at quite similar level (AWCD from 103.60 to 136.83). It is worth noting that some bacterial strains isolated from smelter wasteland such as: LC21—*Pseudomonas sp*., LC17—*Olivibacter soli*, LC8—*Pseudomonas caspiana*, LC7—*Burkholderia sp*., LC6C—*Pseudomonas sp*. were active in utilizing carbon at low pH (pH 5), even though they orginally grew in neutral or slighly alkaline conditions. Soil salinity is one of the most serious and common environmental stresses, which can significantly impair plant production. Some of the tested strains were adapted to a salinity at the level 1% NaCl: 3 of them belonged to *Pseudomonas* and another one was *Olivibacter soli*. This suggests that they would be effective in plant growth in mild saline conditions, for example in over-fertilized soil. It can be assumed that strains that have the ability to metabolize a wide range of substrates and show tolerance to high salinity concentrations and acidic pH could be better adapted to changing environmental conditions. *Olivibacter soli* was also able to grow in media containing up to 4% NaCl, suggesting its ability to tolerate more severe saline conditions. It was, however, less resistant to pH drop. *O. soli* exhibited medium intensity of such mechanisms as ACC, IAA or EPS formation, however under saline conditions this strain can be still potentially valuable promoteur of plant growth. It is worth to note that soil salinity further limits water availability to crops in dry periods. The strains that had the greatest ability to grow on potassium tellurite substrate belonged to *Pseudomonas* and *Burkholderia* genera. The noticeable growth of these bacteria in the presence of such compounds may indicate the resistance to chemical contamination in general and toleration of toxic tellurite^78,79^. The bacterial strains LC17—*Olivibacter soli* and LC6C—*Pseudomonas sp*. also to some extent tolerated sodium butyrate and sodium bromate, respectively. Bacteria that show resistance to sodium bromate, which occurs mainly when ozonation is used to treat water containing bromide (Br-), might potentially become potential candidates for bioremediation processes^80^.

As many scientific studies have shown, the positive effect of plant growth-stimulating bacteria is achieved through a combination of direct and indirect mechanisms, many of which were tested in our study. Direct mechanisms include the solubilization of mineral nutrients such as phosphorus, zinc and potassium, biological N_2_ fixation, the synthesis of phytohormones such as auxins, cytokinins and gibberellins, sequestration of iron by siderophores, and the production of ACC and volatile stimulants growth, i.e. acetoin and 2,3-butanediol, and increasing resistance to stress. Indirect mechanisms include the reduction or prevention of harmful effects caused by pathogenic microorganisms, mainly through the synthesis of antibiotics and/or fungicidal compounds, improving nutrient uptake (e.g. through the production of siderophores), production of hydrogen cyanide (HCN), as well as induced systemic resistance (ISR)^16,56,62,81^.

In general most of strains tested in this study exhibited a range of plant growth promoting mechanisms. Our study enabled clustering bacterial strains with capability to perform certain groups of processes. It is worth emphasizing that strains intensively decomposing carbon organic compounds were also able to produce ACC deaminase that increases stress tolerance in plants, with the example of strain LC6C—*Pseudomonas sp.* Such strains however were less effective in terms of IAA and EPS production, extremely important in promoting plants under insufficient soil moisture. The negative correlation between IAA and ACC deaminase production shall not limit the potential use of the tested strains in crop production as practices helping to adapt to low soil moisture. IAA intensive producers can stimulate root growth which helps plants to reach deeper soil moisture reservoirs. On the other hand ACC deaminase producers can be beneficial in dry periods through enhancing plant tolerance to low moisture stress, additionally offering their effectiveness under other abiotic stresses such as high temperature or soil contamination.

There were also specific strains recognized that exhibit high IAA, biofilm and P solubilization (LC11—*Phyllobacterium sp*.) which makes such strain as especially valuable for regenerative and nature based agriculture. The strain LC8—*Pseudomonas caspiana* was intensively dissolving P while being able also to fix N. This strain therefore can be potentially recommended as a component of biofertilisers offered as alternatives to mineral fertilizers, exhibiting also substantial IAA, EPS and biofilm production that are important traits in plant growth promotion under low moisture stress. LC8—*Pseudomonas caspiana*, LC11—*Phyllobacterium sp*., and LC7—*Burkholderia sp*. that exhibited substantial biofilm and EPS production in the laboratory tests, were further tested in the pot study. The wheat performance confirmed that the mechanisms observed in vitro can be for some strains translated into plant growth promotion, both under optimal moisture conditions and the reduced water availability. It is worth highlighting that LC7 and LC11 that intensively produced biofilm, in case of LC7 combined with high ACC formation, were effective in plant growth promotion under deficit of water. LC8 produced biofilm less intensively in the laboratory conditions but exhibited some salt tolerance also helped the plants to produce high biomass under low moisture.

It should be tested in future experiments whether application of these strains to soil in co-formulation would enhance or rather reduce their plant growth promotion effect. This was not covered by the presented study.

The study based on extensive laboratory measurements and subsequent testing of selected strains in the pot study has obviously some limitations from the agricultural use perspective. Before final protocols for application of the tested strains in crop production are developed, the planned research involves final genetic profiling of bacteria, testing what is the most effective way to deliver strains to a rhizosphere and a final confirmation of their effectiveness in real field conditions. Some organic materials, such as digestate or biochar, are considered as potential carriers of the strains. Such solutions will be tested in next greenhouse experiments to develop the most efficient protocol, before the technology is validated in a field. The study proved that industrial sites such as long-term reclaimed smelter waste piles constitute the habitat of diverse microorganisms that developed a range of processes that potentially might help crops to grow both under optimal and harsh conditions. The microbiome of the reclaimed industrial site has evolved under difficult conditions such as frequent drought, deficiency of nutrients, salinity and soil contamination. These conditions might have contributed to the development of a wide selection of plant growth promotion mechanisms and bacteria adaptability to non-optimal conditions and their survival capacity.

## Conclusions

The study confirmed the hypothesis that bacterial strains isolated from the long-term contaminated site exhibit key plant growth promoting traits. It proves that such extreme sites as tested in our study can be a source of many promising strains from a perspective of developing biofertilisers. Most of tested bacteria exhibited multiple plant growth promotion mechanisms but diversity of processes offered by the tested strains was also observed. This can be obviously translated into a range of biofertilizer products and applications. Such products can be designed to current challenges in agriculture or remediation: drought, salinity, low phosphorus use efficiency and in some cases soil contamination.

The analytical approach performed in our study seems to be an effective way to screen bacterial strains isolated from industrial sites for specific purposes. Considering particular strains tested, *Burkholderia sp*. exhibited mechanisms key from a perspective of managing drought affected or contaminated soils since it produces biofilm and EPS that stabilizes biofilms. *Phyllobacterium sp*. turned out to be high IAA and biofilm producer, and P solubilizer whereas *Pseudomonas caspiana* was intensively dissolving P while being able to fix N. All three isolates confirmed plant growth promotion capability in the greenhouse biotest.

Strains actively producing ACC deaminase that increases stress tolerance in plants, were in general less active in IAA and EPS production. However both groups of bacteria can be likely effective components of bioferilisers applied under insufficient soil moisture stress.

It should be noted that strains preliminarily identified as belonging to the same genera exhibited diverse processes, meaning that the potential uses of these strains cannot be assigned based on taxonomic affiliation. Any strain isolated from such a site should be subjected to a range of tests to identify its potential uses. Further studies shall involve testing the selected promising strains in wider greenhouse and field experiments simulating or reflecting real conditions of plant growth, including experiments conducted under stress conditions (for example drought, salinity, deficiency of nutrients). The effectiveness of the strains applied in consortia shall be also studied and optimal carriers of the bacteria designed.

## Supplementary Information

Below is the link to the electronic supplementary material.

Supplementary Material 1

## Data Availability

The datasets generated during the current study are available in the GeneBank repository, [https://www.ncbi.nlm.nih.gov/genbank/, under the following accession numbers: PP529722, PP529723, PP529724, PP529725, PP529726, PP529727, PP529728, PP529729, PP529730, PP529731, PP529732, PP529733, PP529734, PP529735, PP529736].
