# Supplementary material for: Plant growth promotion mechanisms of bacteria isolated from a long-term reclaimed smelter waste deposit
Source: Sci Rep. 2025 Oct 30;15:38040. doi: 10.1038/s41598-025-21980-w (PMC12575740; doi:10.1038/s41598-025-21980-w)
Supplement: Supplementary file 1 — Supplementary Material 1 [file 41598_2025_21980_MOESM1_ESM.docx]

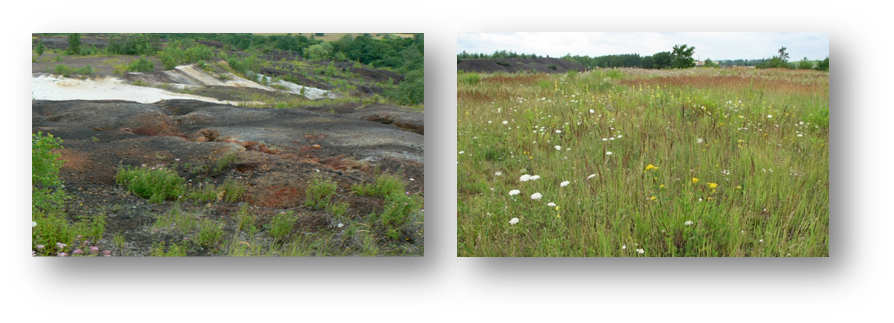


Figure S1. Smelter wasteland in Piekary Śląskie before (left) and after reclamation (right) - the site used for the strain collection

Table S1. Growth efficiency of the tested bacteria across various media

| Isolate no. | Pikovskaya's Broth  (Medium) | TSA medium | LB Medium | Growth after freeze-drying on TSA medium |
| --- | --- | --- | --- | --- |
| LC6B | ++^1^ | ++ | ++ | - |
| LC6C | ++ | ++ | ++ | - |
| LC7 | +++ | +++ | +++ | +++ |
| LC8*^2^ | +++ | +++ | +++ | +++ |
| LC9 | +++ | +++ | +++ | +++ |
| LC11 | +++ | +++ | +++ | +++ |
| LC12 | +++ | +++ | +++ | +++ |
| LC13 | +++ | +++ | +++ | +++ |
| LC14* | +++ | +++ | +++ | +++ |
| LC16 | +++ | +++ | +++ | +++ |
| LC17 | +++ | +++ | +++ | +++ |
| LC18* | +++ | *+++* | *+++* | *+++* |
| LC19 | +++ | *+++* | *+++* | *+++* |
| LC21 | +++ | *+++* | *+++* | *+++* |
| LC22* | +++ | *+++* | *+++* | *+++* |

^1^ - no growth of bacteria

+ low colony growth of bacteria

++ medium colony growth of bacteria

+++ high colony growth of bacteria

^2^ asterix means fast and abundant colony growth (visible already after 1 day)


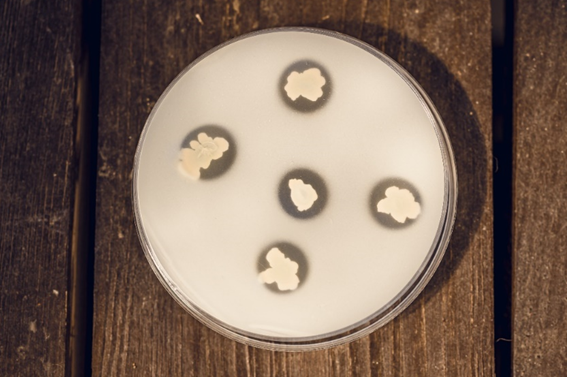


Figure S2. Plate assay showing phosphate solubilizing bacteria (PSB) isolated from smelter of wasteland (single colonies of one bacterial strain)


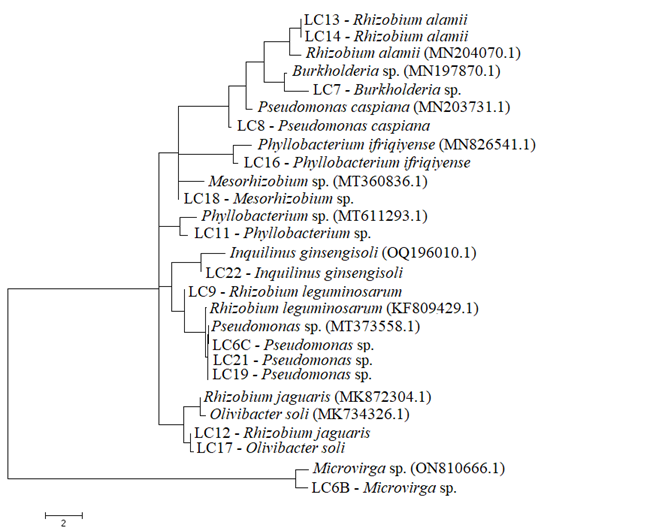


Figure S3. Phylogenetic tree of the tested isolates.


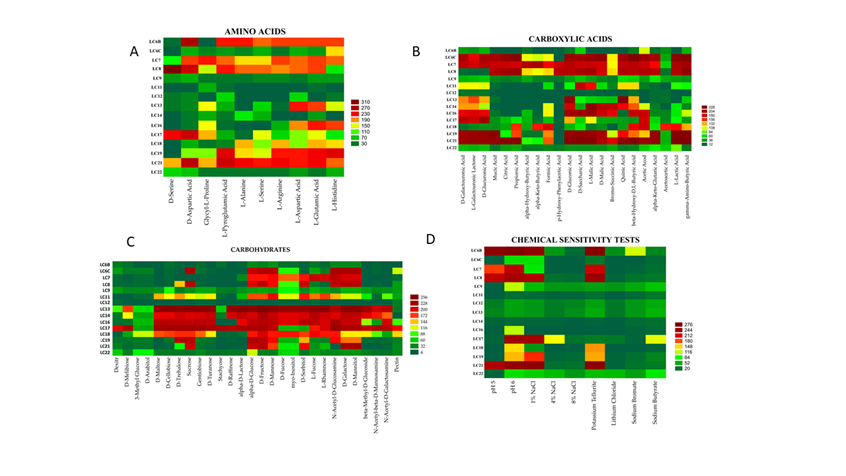


Figure S4. Heatmap of 15 strains showing metabolic profiles for amino acids (A), carboxylic acids (B), carbohydrates (C), and for selected tests of chemical sensitivity (D) after 168 h of incubation. The relative use of selected substrates is depicted by color intensity, based on the legend next to the figure. The highest and lowest consumption rates are indicated by red, orange, yellow and green colors, respectively.

Table S2. Spearman correlation coefficients for relationships between microbiological parameters (phosphate solubilization index – PSI, IAA production, AWCD, nitrogen fixation – NF, ACC deaminase activity, biofilm production – B, EPS production) across 15 tested strains

| IAA | 0.000 |  |  |  |  |  |
| --- | --- | --- | --- | --- | --- | --- |
| AWCD | 0.089 | -0.418**^1^ |  |  |  |  |
| NF | 0.100 | 0.368* | 0.307 |  |  |  |
| ACC | 0.225 | -0.481*** | 0.419*** | 0.070 |  |  |
| B | -0.393* | 0.332* | -0.418* | -0.014 | -0.102 |  |
| EPS | -0.145 | -0.014 | -0.474** | -0.374* | 0.089 | 0.329* |
|  | PSI | IAA | AWCD | NF | ACC | B |

^1^*** significant at 0.001 probability level

** significant at 0.01 probability level

* significant at 0.05 probability level


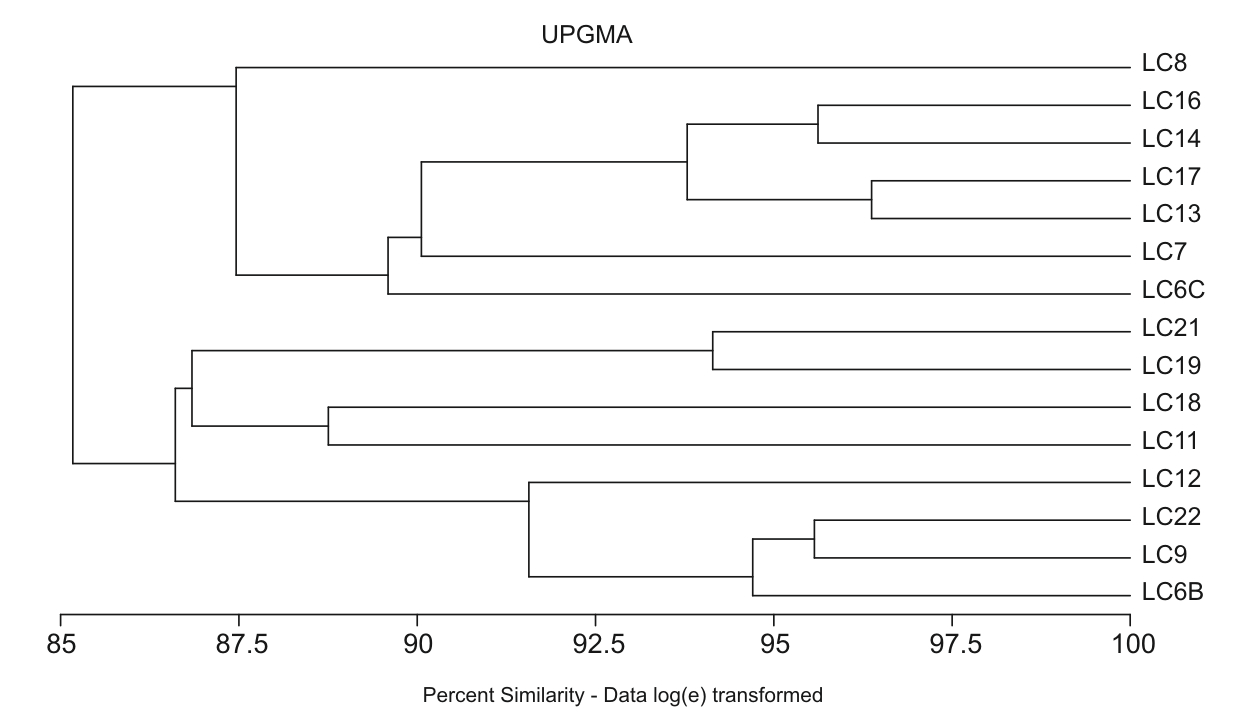


Fig. S5. Clusters of strains with similar profile of biochemical activities
